# Structure determination of organic compounds by a fit to the pair distribution function from scratch without prior indexing

**DOI:** 10.1107/S1600576721002569

**Published:** 2021-05-09

**Authors:** Carina Schlesinger, Stefan Habermehl, Dragica Prill

**Affiliations:** a Institut für Anorganische und Analytische Chemie, Goethe Universität, Max-von-Laue-Strasse 7, Frankfurt am Main, 60437, Germany

**Keywords:** pair distribution function analysis, structure determination, total scattering technique, similarity measures, PDF-Global-Fit

## Abstract

A method for the *ab initio* crystal structure determination of organic compounds by a fit to the pair distribution function (PDF), without prior knowledge of lattice parameters and space group, has been developed. The method is called ‘PDF-Global-Fit’ and is implemented by extension of the program *FIDEL* (fit with deviating lattice parameters). The structure solution is based on a global optimization approach starting from random structural models in selected space groups. No prior indexing of the powder data is needed. The new method requires only the molecular geometry and a carefully determined PDF. The generated random structures are compared with the experimental PDF and ranked by a similarity measure based on cross-correlation functions. The most promising structure candidates are fitted to the experimental PDF data using a restricted simulated annealing structure solution approach within the program *TOPAS*, followed by a structure refinement against the PDF to identify the correct crystal structure. With the PDF-Global-Fit it is possible to determine the local structure of crystalline and disordered organic materials, as well as to determine the local structure of unindexable powder patterns, such as nanocrystalline samples, by a fit to the PDF. The success of the method is demonstrated using barbituric acid as an example. The crystal structure of barbituric acid form IV solved and refined by the PDF-Global-Fit is in excellent agreement with the published crystal structure data.

## Abstract 2

A new automated method to solve organic crystal structures from scratch by a fit to the pair distribution function, without prior knowledge of lattice parameters and space group, has been developed.

## 1. Introduction: PDF on the rise   

Structure determination is an important step in the investigation of molecular solids due to the correlation of the molecular arrangement within the crystal and solid-state properties, such as physico-chemical stability, solubility, bio­availability, and optical and magnetic properties. Knowledge of the crystal structure is crucial to explain or predict these physical and chemical properties (Hata *et al.*, 2020 ▸), as well as to optimize them in terms of crystal engineering (Desiraju, 2003 ▸; Schmidt *et al.*, 2007 ▸). The average crystal structure can be determined by single-crystal analysis or structure determination from powder diffraction data (SDPD) (David *et al.*, 2002 ▸).

Recently, there has been growing interest in the knowledge of the local structure. The local structure may deviate from the average crystal structure (Aksel *et al.*, 2013 ▸), especially for complex materials such as pharmaceuticals (Moore *et al.*, 2009 ▸; Terban *et al.*, 2020 ▸), metal–organic frameworks (Mazaj *et al.*, 2016 ▸), organic pigments (Hunger & Schmidt, 2018 ▸; Schlesinger *et al.*, 2020 ▸), catalysts or magnetic materials, such as semiconductors (Frandsen *et al.*, 2016 ▸). Disorder, lattice defects or surface effects result in a local structure which differs from the average structure found by classical structure determination methods (Proffen *et al.*, 2003 ▸; Young & Goodwin, 2011 ▸). Disorder, for example, can strongly influence the solid-state properties [see *e.g.* Gorelik *et al.* (2016 ▸) and Lindahl Christiansen *et al.* (2020 ▸)]. Therefore, the determination of the local structure of crystalline materials is important for the investigation and development of new materials.

Moreover, the local structure becomes fundamental if no average crystal structure can be determined as, for instance, in poorly crystalline, nanocrystalline solids, as well as for glasses and liquids. In these cases, classical structure determination methods such as single-crystal analysis and SDPD fail (Fernandes *et al.*, 2007 ▸; Dinnebier & Billinge, 2008 ▸; Schlesinger *et al.*, 2019 ▸). Due to their low crystallinity and small domain sizes a reliable indexing of the powder data is not possible. Alternatively, a structure solution from scratch by the global optimization approach of the commercially available software *FIDEL* can be performed, where large sets of trial structures are fitted to the powder pattern without the need for prior indexing (Habermehl, Schlesinger & Schmidt, 2021 ▸). However, while exploring the limits of structure fitting to low-quality powder patterns, this approach requires a certain minimum of crystallinity and long-range order to be successful. This general limitation applies to any variable-cell direct-space method for SDPD that could be performed, *e.g.* the VARICELLA approach (Rapallo, 2009 ▸). If indexing fails, potential lattice parameters and possible space group(s) can be obtained, *e.g.* by a time-consuming crystal structure prediction (Bardwell *et al.*, 2011 ▸; Neumann *et al.*, 2015 ▸), although the comparison of the simulated powder patterns of the predicted crystal structures with the experimental powder pattern can only lead to the average crystal structure (Mörschel & Schmidt, 2015 ▸).

A reliable method to investigate the local structure, *i.e.* short-range ordering, is the pair distribution function (PDF), which can be seen as the probability 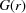 of finding pairs of atoms separated by a distance *r* (Neder & Proffen, 2008 ▸; Young & Goodwin, 2011 ▸; Egami & Billinge, 2012 ▸). The PDF describes the deviation of the microscopic pair density 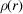 from the average number density 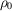 [equation (1)], summed over all atom–atom pairs and weighted with the scattering power of the atoms. The PDF is a total scattering technique, *i.e.* it uses not only the Bragg peaks but also the total powder pattern including the diffuse scattering. 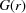 is calculated from carefully measured and background-corrected diffraction data by Fourier transformation of the corrected and normalized coherent scattered intensity 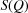 of the sample [equation (1)], *Q* [equation (2)] being the magnitude of the scattering vector, with θ the scattering angle and λ the wavelength of the used radiation (Egami & Billinge, 2012 ▸):
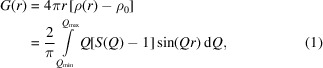


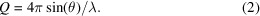



The classical application of PDF analysis entails qualitative and quantitative phase analysis (Zea-Garcia *et al.*, 2019 ▸), including the determination of the domain size of nanoparticles (Neder & Korsunskiy, 2005 ▸) or the amorphous content of the sample (Peterson *et al.*, 2013 ▸). The PDF is frequently used to study the local structure of inorganic materials, liquids and glasses (Juhás *et al.*, 2010 ▸; Young & Goodwin, 2011 ▸; Ojovan & Louzguine-Luzgin, 2020 ▸). While the PDF analysis of inorganic compounds has been steadily developed, the PDF analysis of organic compounds has been slightly delayed. The reasons for this are manifold and include, among other things, the low scattering power of mainly carbon and hydrogen atoms, as well as the different PDF peak widths caused by intermolecular versus intramolecular atom pairs (Rademacher *et al.*, 2012 ▸; Prill *et al.*, 2015 ▸). However, the number of organic materials investigated by PDF analysis is rapidly rising due to the growing interest in their local structure (Bates *et al.*, 2006 ▸; Davis *et al.*, 2013 ▸; Billinge, 2015 ▸; Terban *et al.*, 2016 ▸, 2020 ▸; Rantanen *et al.*, 2018 ▸). Several advances in local structure investigation by a fit to the PDF have been published. However, these methods – regardless of whether an organic or inorganic sample is investigated – require at least a rather well matching crystal structure model(s) (Farrow *et al.*, 2007 ▸; Neder & Proffen, 2008 ▸; Yang *et al.*, 2020 ▸) or at least the knowledge of the unit cell and space group (Prill *et al.*, 2016 ▸) in order to succeed in a reasonable fit. Remarkable work was recently published describing the determination of the space group from the PDF data (Liu *et al.*, 2019 ▸). Nevertheless, the identification of the lattice parameters is challenging for nanocrystalline compounds and often ends without an outcome. Hence, a new method is required to determine the local structure without prior indexing. Such a new method, the PDF-Global-Fit, is presented here. Its aim is to solve the local structure of organic compounds from scratch by a fit to PDF data, without prior knowledge of lattice parameters and space group.

## 2. Method development: structure determination by a fit to the PDF   

**Figure 1:**
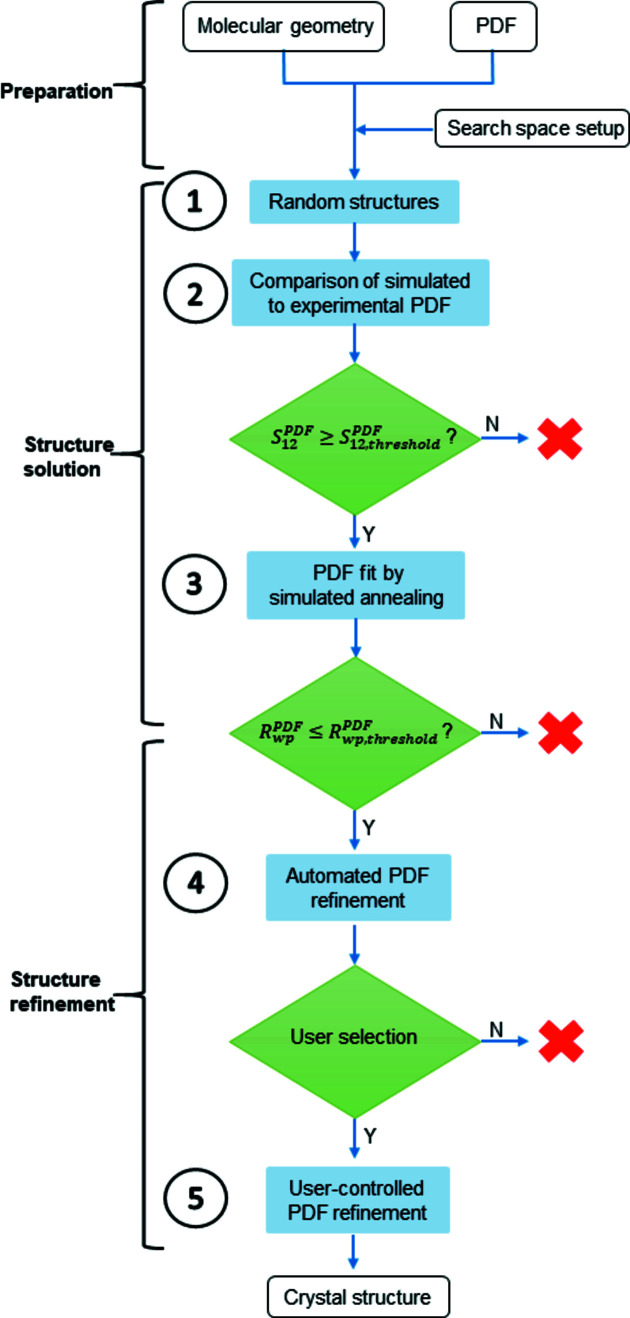
Flowchart of the new structure solution method by a fit to the PDF in general. The encircled numbers define the steps 1–5 of the PDF-Global-Fit. Y = yes, N = no.

The general procedure of the PDF-Global-Fit is shown in Fig. 1 ▸. Only two files are needed as input, *i.e.* a carefully determined experimental PDF and a molecular geometry. An initial molecule model can be taken from an already solved crystal structure of a known polymorph or similar compound, or alternatively derived by a geometry optimization using quantum-mechanical (QM) or force-field methods. Since the PDF-Global-Fit is designed to solve the structure of nanocrystalline substances of hitherto-unknown crystal structures, the QM geometry-optimized molecular model has been used as a start for the development.

The structure solution is based on trial structures generated with the *FIDEL* software (Habermehl *et al.*, 2014 ▸; Habermehl, Schlesinger & Schmidt, 2021 ▸). For this purpose, a reliable search-space setup is needed; a selection of investigated space groups and possibly special positions of the molecule, reasonable ranges for the lattice parameters and the cell volume, and if required the selection of internal degrees of freedom have to be defined in the preparation. According to the search-space setup the trial structures are generated with random values for the lattice parameters *a*, *b*, *c*, α, β, γ, the fractional molecular position *m*_*x*_, *m*_*y*_, *m*_*z*_ and the molecular orientation φ_*x*_ , φ_*y*_, φ_*z*_, as well as possibly random values for selected intramolecular degrees of freedom. All randomly created structural models that are outside the user-defined unit-cell-volume range are discarded. Moreover, only random structures that do not exhibit any kind of molecular overlap are considered.

The PDF-Global-Fit consists of five steps. The generated trial structures (step 1) are subjected to two subsequent structure solution steps: a comparison of the simulated PDF of the structural model with the experimental PDF (step 2) is followed by a fit of the structural model to the experimental PDF (step 3).

In step 2 the simulated PDF is compared with the experimental one by calculation of the similarity measure 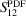 introduced by Habermehl, Schlesinger & Prill (2021 ▸), which is based on the generalized similarity measure using cross-correlation functions according to de Gelder *et al.* (2001 ▸). The random structures are ranked by the similarity. All structures that do not reach a given minimum similarity (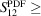 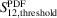; *e.g.* 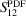 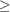 0.8) are discarded.

In step 3, the remaining structure solution candidates are fitted to the experimental PDF curve using the program *TOPAS Academic 6* (Coelho *et al.*, 2015 ▸; Coelho, 2018 ▸), which is called by *FIDEL*. This structure fitting is a restricted simulated annealing (SA) structure solution approach provided by *TOPAS* (Coelho, 2000 ▸; Coelho *et al.*, 2015 ▸).

At the end of step 3, the optimized structure candidates from the SA fit are ranked by their 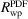 value and only those structural models that exhibit the lowest 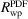 value (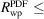 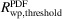; *e.g.* 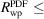 35%) are considered further. The complete structure solution process is automated by *FIDEL*.

In step 4, the remaining structural models are subjected to an automated structure refinement against the experimental PDF using *TOPAS*.

In step 5 a user-controlled refinement of the best structure candidate, or in case of ambiguity several promising candidates, to the PDF data with *TOPAS* is performed.

The *TOPAS* input files for structure solution and refinement were based on the technical references and examples provided with the *TOPAS Academic 6* software (Coelho *et al.*, 2015 ▸; Coelho, 2016 ▸).

### 2.1. Search-space setup and generation of the random structures (step 1)   

The choice of investigated space groups is usually based on the statistics of space-group frequencies according to the molecular symmetry (Pidcock *et al.*, 2003 ▸; Pidcock & Motherwell, 2004 ▸). This means that the most frequent combinations of space group and Wyckoff position of the molecule are considered. Hence, molecules of *C*_1_ or any higher point-group symmetry have to be investigated in selected space groups with the molecule on a general position and *Z*′ = 1. If the molecule belongs to a higher-symmetry point group, in particular if it has an inversion centre, the selection based on frequency statistics will also include the investigation of certain space groups in combination with *Z*′ < 1 and the molecule on a special position. The space groups in which possibly isomorphic or chemically similar compounds crystallize should also be considered. The selection and number of space groups is the user’s decision, considering the available computational resources. If the initial selection does not lead to satisfactory results, additional calculations should be performed in less frequent space groups and/or with *Z*′ > 1 (*e.g.* in space group *P*1 with *Z*′ = 2, which also covers space groups of higher symmetry). For each selected combination of the space group and the general or special position of the molecule, a large set of trial structures is generated, with random values for (at most) the following parameters: the lattice parameters *a*, *b*, *c*, α, β, γ, molecular position *m*_*x*_, *m*_*y*_, *m*_*z*_, molecular orientation φ_*x*_, φ_*y*_, φ_*z*_ and selected intramol­ecular degrees of freedom.

The lattice parameter ranges are set according to the size of the molecule (Pidcock & Motherwell, 2004 ▸). The minimal unit-cell lengths were set to 3 Å, corresponding to the typical π–π-stacking distance. The maximal unit-cell lengths were set on the basis of the longest intramolecular atom–atom distance in the molecular model, taking into account the van der Waals radii and an additional increment of 0.3 Å. The upper boundaries of the cell lengths were derived by multiplying the maximal value for one molecule by the number of molecules in each unit-cell direction according to the space-group symmetry. For the molecules that exhibit many different conformations, which cannot be easily predicted, the largest possible intramolecular atom–atom distance should be taken as a longest possible intramolecular distance. Therefore, the unit cell is large enough for every possible conformation that could occur.

The cell volume is restricted to a certain range to avoid intermolecular contacts which are too close and unreasonable voids. Sensible ranges for cell volumes are derived using increment systems, *e.g.* Hofmann’s volume increments (Hofmann, 2002 ▸), and/or known crystal structures of similar substances, chemical derivatives, other polymorphic forms or solvates, *e.g.* extracted from a suitable database such as the Cambridge Structural Database (Allen & Motherwell, 2002 ▸).

The position and orientation of the molecules in the random structural models are basically unrestricted. However, these parameters are chosen from ranges according to the space-group symmetry (*e.g.* inside the asymmetric unit) in order to avoid an excess of redundant or impossible representations. Furthermore, no trial structure that exhibits unreasonable interatomic distances, *i.e.* molecular overlap, is considered.

### 2.2. Simulation and comparison of PDF curves from structural models (step 2)   

A PDF curve 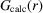 of a given structural model is simulated on the basis of equation (3),[Disp-formula fd3] including the interatomic distance *r*, the scattering powers 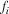, 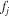 of the atoms *i*, *j*, 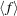 as the average scattering power of the sample and the Dirac delta function 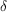 (Egami & Billinge, 2012 ▸):
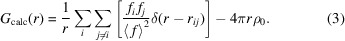



The simulation of the PDF can be performed either using *TOPAS* (Coelho, 2018 ▸) automatically invoked by *FIDEL* or using the *libdiffpy* library of *DiffPy-CMI* (Juhás *et al.*, 2015 ▸) implemented as part of *FIDEL*. They both use constant scattering powers evaluated at the *Q* value of zero for 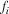, 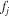 in equation (3), corresponding to the atomic number for a neutral atom. Alternatively, the calculation of the PDF from a structural model could be done via reciprocal space, taking into account the *Q* dependence of the atomic form factors (Neder & Proffen, 2020 ▸). We used *TOPAS* for PDF simulation, since it was used in the subsequent steps of the overall procedure as well. For the simulation, two different isotropic displacement parameters are used, one for intramolecular distances and one for intermolecular ones (Rademacher *et al.*, 2012 ▸; Prill *et al.*, 2015 ▸). The simulated PDF and the experimental PDF are compared and ranked according to their calculated similarity measure 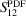 [equation (4)] as implemented in *FIDEL* (Habermehl, Schlesinger & Prill, 2021 ▸). 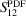 is based on 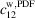 [equation (5)], the integral of the weighted cross-correlation function 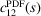 [equation (6)] of the two curves, and normalized by the respective integrals of the weighted auto-correlation functions 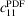 and 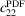:
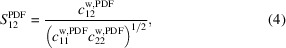


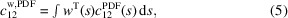


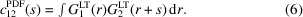



The cross-correlation function 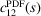 of two PDFs, 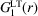 and 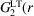), correlates each data point of one curve to the data points at the distance *s* in the other curve [equation (6)]. The acronym LT denotes that the PDF curves are subjected to a linear transformation which shifts 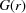 to positive values while keeping a common baseline. By weighting the cross-correlation function with the triangular function 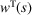 the correlation of data points is restricted to a defined neighbouring range of 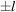 [equation (7)] before integration over all data-point distances within the given range yields 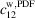 (Habermehl, Schlesinger & Prill, 2021 ▸):
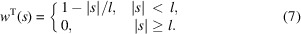



From equation (4) an 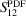 value of 1 implies identity of the two PDF curves. The similarity measure 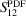 is a powerful tool for the comparison of two roughly matching PDF curves, especially if their signal positions strongly deviate. A comparison based on pointwise differences would in many cases fail to indicate a considerable concordance of the two PDFs, whereas the similarity measure quantifies their congruence sufficiently well (Habermehl, Schlesinger & Prill, 2021 ▸).

The similarity measure 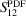 is calculated for all structures. The structures are ranked according to their 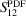 values, and all structures that have a value below a threshold value 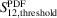 are discarded. 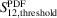 is a user-defined value, which is expected to vary slightly depending on the investigated problem, in particular with respect to the experimental data.

### 2.3. Fit to the experimental PDF by simulated annealing (step 3)   

Trial structures that qualified as structure solution candidates by reaching at least a given similarity threshold value 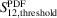 are subjected to a fit to the experimental PDF using the SA method of *TOPAS* (Coelho *et al.*, 2015 ▸) controlled by *FIDEL*.

The agreement of a structural model with the experimental PDF is commonly quantified by a weighted agreement factor 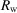 [equation (8)] derived from the pointwise differences between the observed PDF 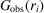 and the calculated PDF 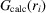 with the corresponding weight 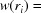 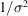 calculated from the error σ of 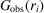 at each data point *i* (Egami & Billinge, 2012 ▸):
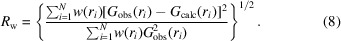



Approaches based on pointwise differences serve well for the comparison and fitting of structural models to the experimental PDF if the model is already close to the best match. However, a pointwise comparison tends to fail or become indecisive if the shifts in signal positions are too big, in particular in the case of lattice parameter deviations (Habermehl, Schlesinger & Prill, 2021 ▸). The SA method of *TOPAS* can very efficiently determine the molecular position and orientation if roughly correct lattice parameters are given. In our experience of organic substances the sum of lattice parameter deviations may not exceed 4–10%; otherwise the SA by a fit to the PDF fails. The robustness against deviating lattice parameters, on the other hand, is a strength of *FIDEL*’s approach based on the similarity measure 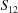. Hence, the hierarchical search strategy of the global optimization by *FIDEL* (Habermehl, Schlesinger & Schmidt, 2021 ▸) has been combined with the SA procedure of *TOPAS* in order to ally the strengths of the two approaches.

The SA fit is performed using basically the same representation of the structure candidates and fitted parameters as described for *FIDEL*, *i.e.* the lattice parameters and the position and orientation of the molecule. The molecular geometry is described by a *z* matrix, which may include distances, angles or dihedral angles corresponding to selected internal degrees of freedom. According to the SA method the molecular position *m*_*x*_, *m*_*y*_, *m*_*z*_ and the molecular orientation φ_*x*_, φ_*y*_, φ_*z*_ are randomized on the basis of the start structure. The initial candidate is a trial structure that had been compared with but not fitted to the experimental PDF before. Hence, during the SA the lattice parameters were allowed to vary within comparably narrow ranges, *e.g.* 5% of the lattice parameters of the initial structure.

The *TOPAS* SA fit is performed by a robust, automated four-step optimization approach. The zero point of the PDF, as well as the scaling factor, are optimized in each step. At first the inter- and intramolecular displacement parameters, the envelope, the molecular position *m*_*x*_, *m*_*y*_, *m*_*z*_, and the mol­ecular orientation φ_*x*_, φ_*y*_, φ_*z*_ are fitted on the basis of the structure candidate. In the second SA step these optimized values are kept fixed during a subsequent fitting of the lattice parameters. In the third SA step a simultaneous fit of the lattice parameters, *m*_*x*_, *m*_*y*_, *m*_*z*_ and φ_*x*_, φ_*y*_, φ_*z*_ is performed. In the last SA step, all mentioned variables are fitted simultaneously to the experimental PDF data. The optimized structures are ranked according to their weighted-pattern *R* value 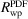 calculated by *TOPAS* as a figure of merit of the fit of the structure candidate to the experimental PDF.

### 2.4. Structure refinements (steps 4 and 5)   

In step 4 the fitted structure candidates from step 3 that yielded 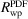 values below a predefined threshold value are refined to the experimental PDF using *TOPAS Academic 6* (Coelho, 2018 ▸). The molecular geometry is described by internal coordinates using the *z*-matrix formalism, optionally including selected degrees of freedom. At first, the lattice parameters, scale factor, zero point, damping of the PDF curve, and one inter- and one intramolecular isotropic displacement parameter were refined simultaneously. Subsequently, the position and orientation of the molecule were refined. Alternatively, the molecular geometry can be refined using fractional atomic coordinates with restraints for bond angles, bond lengths and planar groups.

The results of the automated refinement are evaluated by the user with respect to the 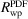 values, the difference curves of the calculated and observed PDF, the molecular packing or hydrogen-bond pattern, and other criteria. On the basis of this thorough evaluation, one or, in the case of ambiguities, several structures are selected for the final user-controlled refinement (step 5).

## 3. PDF-Global-Fit: barbituric acid as an example   

**Figure 2:**
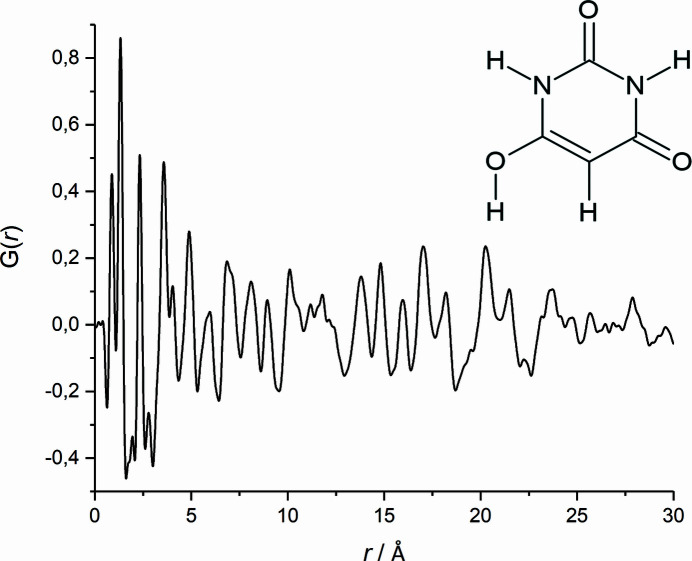
Experimental PDF curve of barbituric acid polymorphic form IV (*Q*_max_ = 21.9 Å^−1^). The inset shows the structural formula of barbituric acid in its enol tautomeric form, which is present in form IV.

For the development and validation of the method a rigid organic compound with a known crystal structure was considered reasonable. Hence, barbituric acid (C_4_H_4_N_2_O_3_, Fig. 2 ▸) was chosen, which is a commercially available, very well known, rigid, organic molecule that contains a small number of atoms. Barbituric acid exhibits keto–enol tautomerism and forms different polymorphs with different tautomers. At ambient conditions, the thermodynamically stable form is polymorph IV, which contains the enol tautomer shown in Fig. 2 ▸. The crystal structure of this polymorph of barbituric acid was solved by X-ray and neutron powder diffraction (Schmidt *et al.*, 2011 ▸), and later confirmed by X-ray single-crystal diffraction (Marshall *et al.*, 2016 ▸). It crystallizes in *P*2_1_/*n* with *Z* = 4 and unit-cell parameters of *a =* 11.87614 (6), *b* = 8.91533 (4), *c* = 4.83457 (3) Å and β = 95.0854 (4)° (Schmidt *et al.*, 2011 ▸). For comparability this crystal structure was transformed to the standard unit-cell setting of *P*2_1_/*c* with *a* = 4.83457, *b* = 8.91533, *c* = 12.4192 Å and β = 107.729°. The crystal structures resulting from the structure determination by a fit to the PDF will be compared with this known crystal structure of barbituric acid in *P*2_1_/*c*.

### 3.1. Experimental detail   

Barbituric acid was purchased from Sigma Aldrich (99% purity) and used without further purification. The sample was milled in a mortar and subsequently placed in a polyimide capillary (1 mm in diameter) which was sealed with clay at both ends. The X-ray powder diagram of the sample was measured at 300 K at the X17A beamline of the National Synchrotron Light Source at Brookhaven National Laboratory. A monochromatic incident X-ray beam conditioned using an Si(311) monochromator to have an energy of 67.42 keV (λ = 0.1839 Å) was used. The 2D PerkinElmer amorphous silicon detector was mounted orthogonally to the beam path with a sample-to-detector distance of 204.2 mm, as calibrated with an LaB_6_ standard sample. Multiple scans were performed to achieve a total exposure time of 30 min. The 2D diffraction data were integrated and converted to intensity versus 2θ using the software *FIT2D* (Hammersley, 2016 ▸). The data were corrected and normalized and then truncated at a finite maximum value of the momentum transfer *Q*_max_, which was optimized to avoid large termination effects whilst maximizing the signal-to-noise ratio, using the program *PDFgetX3* (Juhás *et al.*, 2013 ▸) to obtain the total scattering structure function, *F*(*Q*), and *G*(*r*). The value *Q*_max_ = 21.9 Å^−1^ was found to be optimal for barbituric acid.

The molecular geometry of barbituric acid was calculated by geometry optimization at the B3LYP/6-31g** level using *GAUSSIAN* (Frisch *et al.*, 2009 ▸). Although a high-quality single-crystal structure is available for barbituric acid, the molecular geometry was derived from QM geometry optimization in order to represent a general example for the proof-of-concept evaluation of the PDF-Global-Fit procedure.

All calculations (PDF simulation, structure solution and refinement) were performed on a standard desktop PC running a 64 bit Windows system and equipped with an Intel Core i7-3770 processor and 32 GB RAM. The generation of the random structures and the comparison of the simulated and experimental PDFs (steps 1 and 2) take approximately 4 days. The structure solution and refinement (steps 3 and 4) which take approximately 3 weeks are the most time-consuming steps in the procedure. This is a rather long time; however, the process itself is still in development and calculation steps will be optimized.

### 3.2. Search-space setup for the PDF-Global-Fit   

For the preparation step the PDF data and the *z* matrix of a QM geometry-optimized barbituric acid molecule were provided as input files.

#### 3.2.1. Parameters for the search-space setup (step 1)   

Barbituric acid exhibits the point group *C*_s_. The most likely space groups for barbituric acid were selected according to space-group statistics for organic compounds (Pidcock *et al.*, 2003 ▸; Pidcock & Motherwell, 2004 ▸). To save computational time, three space groups were chosen, which already cover over 75% of all crystal structures with molecules having the molecular symmetry *C*_s_: *P*1, *P*2_1_/*c* and *P* 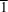 each with *Z*′ = 1. Moreover, the chosen space groups cover various supergroups with higher symmetries. For example, calculations in *P*1, *Z* = 1 can also result in structures in *Pm*, *Z* = 1 and *Cm*, *Z* = 2, calculations in *P*2_1_/*c*, *Z* = 4 include structures in *Pnma*, *Z* = 4 and *P*2_1_/*m*, *Z* = 2 *etc*. The search-space setup is given in Table 1 ▸, including the ranges of lattice parameters and cell volumes allowed. The minimal unit-cell lengths were set to 3 Å, corresponding to the typical π–π-stacking distance. The maximum limits for the unit-cell parameters were derived from the longest intramolecular distance in the geometry-optimized barbituric acid, which is 5.535 Å. After adding the van der Waals radii plus 0.3 Å to avoid close contacts, the maximum space for one barbituric acid molecule in one direction of the unit cell is 8.5 Å. The number of possible molecules in each direction depends on the space group and symmetry operators, *e.g.* in *P*2_1_/*c* a molecule can be situated four times in the *c* direction; therefore the maximum value of *c* is 4 × 8.5 Å = 34 Å.

The estimated molar volume of barbituric acid is 133.57 Å^3^ using Hofmann’s increment system (Hofmann, 2002 ▸). In *P*1 and *P* 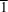 the range for the cell volume was set to ±15% of that value. In *P*2_1_/*c* the minimum cell volume was set to −15%. It is known that due to packing effects the cell volume is overestimated for aromatic planar compounds in higher-symmetry space groups. Hence the maximum cell volume was set to +5% of the Hofmann volume.

#### 3.2.2. Simulation of the PDF curves from structural models (step 2)   

To ensure comparability, the simulations of the PDF curves from the structural models were all performed under the same fixed conditions with respect to the instrumental envelope and the intra- and intermolecular atomic displacement parameters using the program *TOPAS*. The instrumental envelope was determined using a reference substance, resulting in a value of 48.0 Å^−1^. The intramolecular displacement parameter *B*_intra_ of 0.16 Å^2^ was determined using a simulated PDF curve of a single molecule of barbituric acid. For small planar organic compounds, a ratio of *B*_intra_ to *B*_inter_ of 1 to 3.75 was found (Prill *et al.*, 2015 ▸), resulting in an intermolecular displacement parameter *B*_inter_ of 0.6 Å^2^. The simulated PDF curves were calculated and compared with the experimental one in a range of 1–20 Å.

#### 3.2.3. Threshold criteria for the selection of structure candidates (steps 2 and 3)   

During the structure solution process of the PDF-Global-Fit a large set of random structural models within the search-space setup outlined before is incrementally reduced to smaller sets of qualified structure candidates. At two points in the search for a correct local structure representative, promising structural models were selected according to the settings of threshold criteria: the first point was after the comparison step (step 2) and the second point after the SA fit (step 3).

Due to the first criterion the structural models that do not reach a minimal similarity measure value 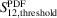, resulting from the comparison of the calculated and the experimental PDF curve, were sorted out. To define the threshold value, preliminary tests were performed on modified crystal structures of barbituric acid and on randomly created structures. Preliminary tests on modified structures of barbituric acid [root-mean-square Cartesian displacement (RMSCD) (van de Streek & Neumann, 2010 ▸) values smaller than 0.25 Å] resulted in values of 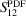 ≥ 0.985 (Habermehl, Schlesinger & Prill, 2021 ▸). Further tests on randomly created crystal structures of barbituric acid showed that the 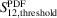 value of 0.985 leads to a reasonable number of structural models in the next step of the structure solution. Therefore, the requested similarity value of 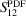 = 0.985, using the neighbourhood range parameter *l* = 0.53 Å, was found to be adequate for the example presented here. Only structure candidates with 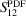 values higher than the threshold criterion were subjected to the SA fit to the experimental PDF data using the *TOPAS* software as described earlier. The second selection step (step 3) was then imposed by discarding all fitted structure candidates that exceed a maximal 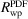 value of 35%.

### 3.3. Results   

A set of 100 000 random structures in each investigated space group was generated in step 1. The numbers of structure candidates qualifying in the subsequent steps 2–5 differ greatly depending on the space group (Table 2 ▸). In *P*2_1_/*c*, 439 structure candidates reached a similarity value 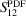 above 0.985 after comparison step 2, whereas no comparably promising structure candidates are observed in *P*1. The three best qualified structure candidates in *P*1 exhibit 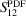 values of about 0.98. Accordingly, no qualified structure candidate was further considered in *P*1. In *P*1 (*Z* = 1), only layered structures with parallel molecules are possible. Apparently, this packing motif is unfavourable for the enol tautomer of barbituric acid.

After the comparison step (step 2) the similarity measures of the four top-ranked candidates in *P* 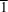 were slightly higher than the best one in *P*2_1_/*c*. The lattice parameters showed an insignificant trend to a small *a* axis (range of 3.3–7.3 Å). By visual inspection of the best structural models it was noted that a criss-cross packing motif is more frequent than other packings, such as layered structures. The best ten structural models for each of the space groups *P*2_1_/*c* and 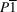 according to the similarity measure from the comparison (step 2) of the simulated PDF curve with the experimental one are shown in Table S1 in the supporting information.

Table 3 ▸ represents the results of the SA fit of the structure candidates to the experimental PDF data, ranked by the 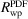 value (step 3). By comparison of the 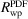 values it was obvious that those of the structure candidates in *P*2_1_/*c* are smaller than the ones from the models in *P* 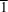, although one model in *P* 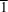 exhibits an 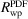 value as low as the structural models in *P*2_1_/*c*. As expected, the spread of the lattice parameters is significantly smaller after structure fitting (step 3) than in the previous step (step 2) of the PDF-Global-Fit and crucial trends were obvious.

**Figure 3:**
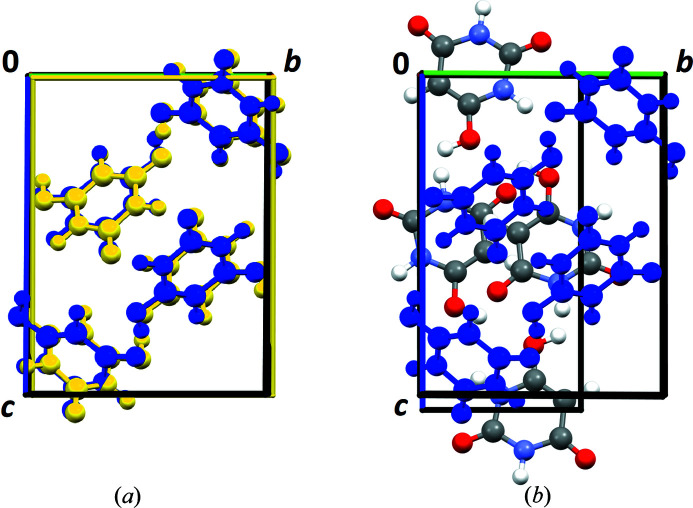
A comparison of the structures after the SA fit (step 3). (*a*) A comparison of the structure candidate No. 54845 (yellow) with the published structure (blue). (*b*) A comparison of structure candidate No. 54100 (colour by elements) with the published structure (blue). View along the *a* axis.

The smallest 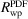 value of 26.6% is significantly lower than all the others. This structure candidate, number 54845 in *P*2_1_/*c*, is, already after step 3, in good agreement with the published structure of barbituric acid form IV: the correct lattice parameters are already found, as well as the correct molecular position, although tiny discrepancies in the molecular orientation are shown, *i.e.* most atomic positions match well [Fig. 3 ▸(*a*)]. Nonetheless, the other structural models in *P*2_1_/*c* also consistently show the correct criss-cross packing motif, although the majority of structure candidates exhibit intermolecular contacts that are too close [Fig. 3 ▸(*b*)].

**Figure 4:**
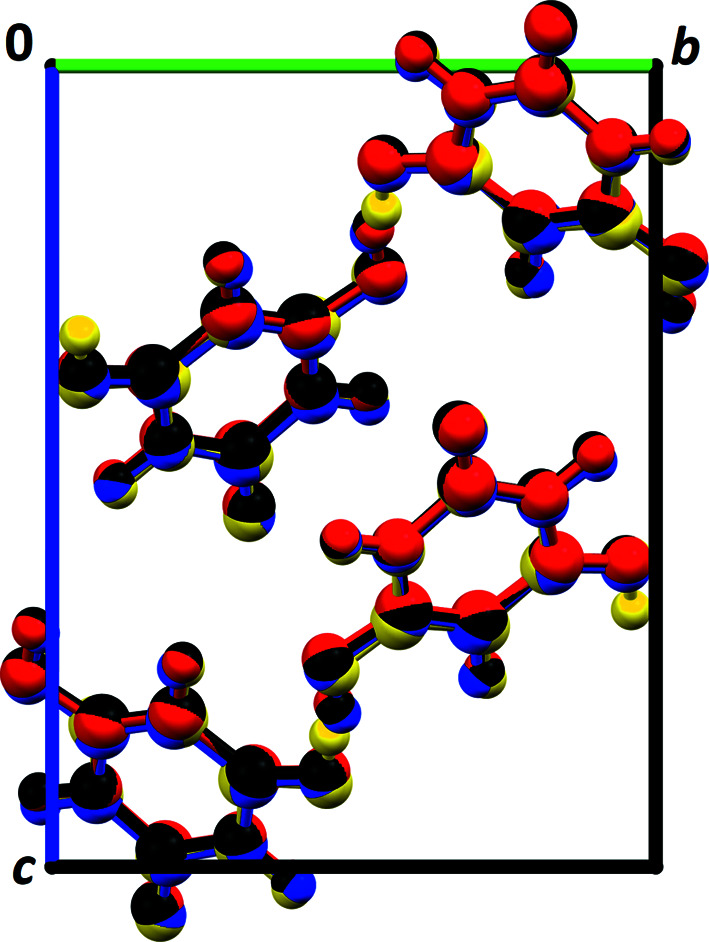
The best three structure candidates found by the PDF-Global-Fit after the user-controlled refinement (step 5): 1 (red), 2 (black) and 3 (yellow) in comparison with the published structure determined by Rietveld refinement (blue) (Schmidt *et al.*, 2011 ▸). View along the *a* axis.

**Figure 5:**
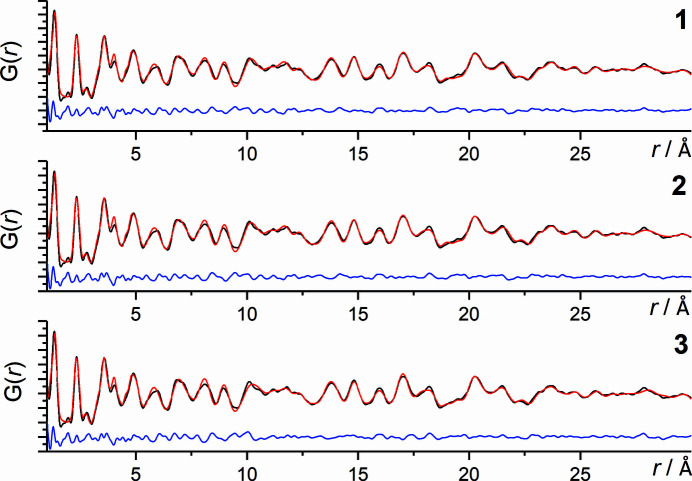
PDF curves of structures 1 (top), 2 (middle), 3 (bottom); experimental PDF (black), calculated PDF (red), difference curve (blue).

As shown in Tables 2 ▸ and 3 ▸, only 11 structure candidates satisfied the 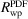 threshold criterion after the SA fit (step 3). For the subsequent automated refinement of these 11 remaining structure candidates to the experimental PDF (step 4) the *r* range for the comparison of simulated and experimental PDF curves was increased to 1–30 Å. The automated refinement was followed by a user-controlled one (step 5). After these two refinement steps three structures (structures 1, 2 and 3) in *P*2_1_/*c* exhibit a 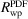 value as low as approximately 20% (Table 4 ▸). The lattice parameters are in perfect agreement with the lattice parameters of the crystal structure published by Schmidt *et al.* (2011 ▸). All structure representatives are chemically sensible, signifying that the structures exhibit no voids within the packing and have a sensible three-dimensional hydrogen-bond network. The correct molecular position was found in all three instances (Fig. 4 ▸). The RMSCD values (van de Streek & Neumann, 2010 ▸) relative to the published structure were calculated for all non-H atoms for these three structures. The corresponding values are 0.049 Å for 1, 0.045 Å for 2 and 0.064 Å for 3. One of the three models (3, yellow model in Fig. 4 ▸) shows a minor deviation of the molecular orientation relative to the published structure: the position of one H atom of the H—O bond is not exact. This corresponds to a molecular orientation switch of 180°. The positions of all the other atoms (nitro­gen, oxygen and carbon) are correct as determined by the PDF-Global-Fit. This is a result of the low scattering power of one H atom (∼0.008%) when compared with the other atoms. Moreover, the determination of hydrogen positions from X-ray diffraction data is challenging, and hence it is conventional to calculate the associated hydrogen positions by a QM or force-field method. Nevertheless, the correct hydrogen-bond network is represented. The 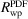 value is as low as for the other two structure representatives and the difference curve of the calculated and observed PDF curves is smooth (Fig. 5 ▸). Thus, structure candidate 3 can also be considered as the correct structure found by the PDF-Global-Fit. Additionally, it cannot be ruled out that the hydrogen position is slightly disordered in the local structure and structure 3 could be an alternative representative for the local structure of barbituric acid.

The evolution of the lattice parameters of the best structure candidate (structure 1) within each step of the PDF-Global-Fit is illustrated in Table 5 ▸ and represents the improved optimization of the structure candidate to the experimental PDF data.

Using barbituric acid as an example, the power of the PDF-Global-Fit without prior indexing using *FIDEL* and *TOPAS* could be demonstrated and highlighted. The correct crystal structure of barbituric acid could be found three times starting from a set of only 300 000 random structures in the three most frequent space groups *P*1, *P*2_1_/*c* and *P* 1 by a fit to PDF data.

## 4. Discussion   

Barbituric acid is a test case, which was used to demonstrate the feasibility and power of the PDF-Global-Fit method. What about more complex structures? Prill *et al.* (2016 ▸) have shown that the structure of the organic compound allopurinol can be successfully solved even in *P*1 with four independent mol­ecules, *i.e.* with 21 degrees of freedom, if the lattice parameters are known in advance. The high information content of PDF data has also been used to determine the local structure of disordered materials, including SF_6_ (Tucker *et al.*, 2007 ▸) and mono­methyl-quinacridone, C_21_H_14_N_2_O_2_ (Schlesinger *et al.*, 2020 ▸). These observations indicate that the PDF data should contain enough information to solve more complex structures than barbituric acid from scratch.

Classical methods for structure determination use the Bragg peaks only. This information is quite limited, especially if the powder pattern contains only a few broad peaks. In contrast, the PDF uses the information from the total scattering, including the diffuse scattering, even in the very high 2θ range, and the background, which is generally ignored by classical structure solution methods.

To estimate the complexity of the structures that in principle should be solvable by the PDF-Global-Fit, a comparison with classical direct-space methods for SDPD might be helpful. Both approaches are based on the information content of the powder diffraction data. Experience shows that the success rate of the direct-space methods is not limited by the size of the molecules, but by the number of degrees of freedom (presupposing that the indexing is reliable). The structure solution by direct-space methods becomes challenging if the number of degrees of freedom (for molecular position, molecular orientation and intramolecular degrees of freedom) exceeds a limit of 20–25 (Florence *et al.*, 2005 ▸; Kabova *et al.*, 2017 ▸; Nilsson Lill *et al.*, 2018 ▸). A similar trend can be expected for the PDF-Global-Fit, given that the unknown lattice parameters increase the number of degrees of freedom.

The advantage of the PDF-Global-Fit in comparison with classical direct-space methods is that no prior indexing is required. Note that the PDF provides the local structure, whereas classical SDPD gives the average long-range ordering in the crystal, which may deviate from the local structure. Therefore, the PDF-Global-Fit can support the classical SDPD for an unindexable powder pattern, such as nanocrystalline samples, but can also be combined with SDPD for crystalline compounds to determine the difference between local and average structure, for example in disordered materials.

The geometrical accuracy of the structures resulting from a fit to the PDF is excellent. The lattice parameters as well as the molecular position and orientation of the investigated compounds determined by a fit to the PDF are in perfect agreement with already published single-crystal data. This observation was made in prior work, where the lattice parameters were (approximately) known, and mainly the mol­ecular position and orientations were determined by a PDF fit (Prill *et al.*, 2016 ▸).

The structure determination of barbituric acid by the PDF-Global-Fit was performed using the PDF only in the range of *r* = 1–20 Å for the structure solution and 1–30 Å for the structure refinement. Actually, the PDF contains signals up to much larger *r* values, because the ordering length (domain size) in the investigated sample is more than 300 Å. The fact that a range of 1–30 Å was fully sufficient for structure determination reveals that the PDF-Global-Fit should also work successfully for nanocrystalline compounds with small domain sizes (*e.g.* 30–100 Å). Hence, the PDF-Global-Fit is a new method for the determination of crystal structures of nanocrystalline compounds from scratch, without the need to index the powder pattern. The PDF-Global-Fit is built on the global optimization method of *FIDEL*, which has been developed and successfully applied for structure determination from unindexed powder patterns of very low, but still sufficient, quality for SDPD (Habermehl, Schlesinger & Schmidt, 2021 ▸). The basic concepts of the approach could be successfully adapted and applied to the structure determination by a fit to the PDF. Other methods to determine crystal structures of nanocrystalline organic compounds include electron diffraction or crystal structure prediction, in combination with X-ray powder diffraction to select the actual structure from the simulated ones. However, the characteristics of all these methods are different. The PDF-Global-Fit is the only method that yields the local structure from the diffraction data, instead of the average structure. Furthermore, the PDF-Global-Fit is the only method that can be applied if the powder pattern contains no Bragg peaks, but only broad humps. (A ‘crystal’ consisting of 5 × 5 × 5 unit cells does not produce any useful Bragg peaks, but provides a reliable PDF.) Of course, a combination of different approaches is also useful.

A second reason as to why the PDF range used for structure solution was restricted to 1–20 Å instead of a broader range, *e.g.* 1–100 Å, is the required computational time. The most time-consuming task of the structure solution is the simulation of the PDFs from structural models, with the time required for the calculation of a single PDF growing roughly proportional to *r* ^3^. This affects the screening of a huge number of trial structures by comparison with the experimental PDF and even more the fitting of structural models. Hence, the restriction of the *r* range is crucial for the feasibility of the structure solution. Although performed with a restricted *r* range, the structure fitting by SA (step 3) still required about 50% of the total computing time of the entire PDF-Global-Fit. Because of the high computing effort in this step, it would be practically impossible to fit all random structures from step 1 to the PDF data (step 3). Hence an adequate reliable preselection of promising structure candidates is unavoidable. The preselection is done by the similarity measure in step 2. This highlights the essential role of two major concepts of the global optimization approach of *FIDEL* for the success of the global fit to PDF or powder patterns: (i) The use of the similarity measure *S*_12_ and its adaptability by variation of the neighbourhood range parameter *l* provides the basis for a comparison of simulated and experimental data that enables the detection of a rough match, in particular with respect to strongly deviating lattice parameters. (ii) From the characteristics of the similarity measure, an effective incremental search strategy can be designed which makes a global fit starting from a huge number of random structures feasible by minimization of the computing time required.

## 3.8. Conclusion   

A novel method called the PDF-Global-Fit is reported for solving organic crystal structures from scratch by a fit to the pair distribution function without prior indexing. Only the molecular geometry and experimental PDF data must be provided as input. The method contains an automated structure solution procedure, according to the Monte Carlo approach, in selected space groups, using the program *FIDEL*. The PDF calculation and the fitting of the structural models are performed using *TOPAS*. Subsequently, a user-controlled refinement of the most promising structure candidates to the PDF data results in the final structure. The suitability of the method was proven using barbituric acid as an example. This is the first time that an organic crystal structure has been solved from scratch by a fit to the PDF without lattice parameters and the space group as input. The implementation of the PDF-Global-Fit in *FIDEL* is still under development and therefore not yet available in a commercial version of the software.

The next steps will be the examination and development of the method, *e.g.* for crystal structures containing molecules with conformational degrees of freedom, nanocrystalline samples, or more complex systems such as hydrates, solvates, salts and cocrystals. Additionally, the procedure has to be further optimized to reduce the computational time in order to gain a higher throughput. Another perspective will be the combination of the fit to the PDF with the fit to the powder pattern under the common framework of the global optimization approach of *FIDEL*.

Nevertheless, the possibility to solve crystal structures from unindexable powder data by a fit to the PDF, or even to obtain the local structure of nanocrystalline organic materials, is within reach.

## Supplementary Material

Supporting information, Table S1. DOI: 10.1107/S1600576721002569/vk5045sup1.pdf

## Figures and Tables

**Table 1:** Search-space setup for the generation of the random structures (step 1) of barbituric acid in *P*1, *P*2_1_/*c* and *P* \overline{1}

	*P*1	*P*2_1_/*c*	*P* \overline{1}
*Z*′	1	1	1
*Z*	1	4	2
*a*_min_ (Å)	3.0	3.0	3.0
*a*_max_ (Å)	8.5	17.1	17.1
*b*_min_ (Å)	3.0	3.0	3.0
*b*_max_ (Å)	8.5	34.1	17.1
*c*_min_ (Å)	3.0	3.0	3.0
*c*_max_ (Å)	8.5	34.1	17.1
α_min_ (°)	90	90	90
α_max_ (°)	120	90	120
β_min_ (°)	90	90	90
β_max_ (°)	120	120	120
γ_min_ (°)	60	90	60
γ_max_ (°)	120	90	120
*V*_min_ (Å^3^)	114	458	227
*V*_max_ (Å^3^)	154	561	307

**Table 2:** Number of structural candidates after each discrimination step during the PDF-Global-Fit; the correct structure was found three times

	*P*1	*P*2_1_/*c*	*P* \overline{1}
Number of random structures (step 1)	100 000	100 000	100 000
Comparison: {S}_{12}^{\rm PDF} \ge 0.985 (step 2)	0	439	223
SA fit: {R}_{\rm wp}^{\rm PDF} \le 35% (step 3)	0	5	6
Correct structures (step 5)	0	3	0

**Table 3:** Best structure candidates of barbituric acid after SA fit (step 3) to the experimental PDF ({R}_{\rm wp}^{\rm PDF} \le 35%)

Structure No.	Space group	{R}_{\rm wp}^{\rm PDF} (%)	*V* (Å^3^)	*a* (Å)	*b* (Å)	*c* (Å)	α (°)	β (°)	γ (°)
54845	*P*2_1_/*c*	26.603	508.33	4.9460	8.9646	12.0907	90	108.527	90
54100	*P*2_1_/*c*	31.317	449.46	6.8661	5.6619	11.8345	90	102.340	90
81533	*P*2_1_/*c*	31.857	498.82	7.5264	5.0621	13.5931	90	105.581	90
76224	*P*2_1_/*c*	32.962	490.84	4.0256	6.0210	20.5214	90	99.331	90
86558	*P*2_1_/*c*	34.097	480.24	6.9655	11.8597	5.9876	90	103.892	90

4150	*P* \overline{1}	32.315	230.65	4.0937	4.9067	11.6193	94.177	88.769	97.750
54062	*P* \overline{1}	33.614	231.80	3.3653	6.0213	11.5356	95.017	95.211	90.957
68229	*P* \overline{1}	33.727	243.66	4.0585	4.9878	12.2882	98.708	90.295	97.621
51118	*P* \overline{1}	34.002	220.70	3.4546	5.6754	11.7294	98.876	91.947	103.134
15782	*P* \overline{1}	34.252	244.00	4.0438	5.0017	12.2898	82.221	89.913	82.205
19486	*P* \overline{1}	34.625	230.04	3.9995	4.9812	11.7673	94.753	95.834	97.585

**Table 4:** The lattice parameters of the best three structure candidates found by the PDF-Global-Fit after the user-controlled refinement (step 5) in comparison with the published structure determined by Rietveld refinement (Schmidt *et al.*, 2011 ▸)

Structure	Space group	{R}_{\rm wp}^{\rm PDF} (%)	*V* (Å^3^)	*a* (Å)	*b* (Å)	*c* (Å)	α (°)	β (°)	γ (°)
1	*P*2_1_/*c*	19.57	512.062 (1)	4.8439 (16)	8.929 (3)	12.423 (3)	90	107.634 (19)	90
2	*P*2_1_/*c*	19.68	512.329 (1)	4.8439 (15)	8.929 (3)	12.429 (3)	90	107.627 (19)	90
3	*P*2_1_/*c*	20.12	511.496 (1)	4.8405 (15)	8.931 (3)	12.417 (4)	90	107.66 (2)	90
Published structure	*P*2_1_/*c*	–	509.867 (5)	4.8346 (3)	8.915 (4)	12.419 (6)	90	107.729 (4)	90

**Table 5:** Evolution of the lattice parameters of structure candidate 1 in *P*2_1_/*c*, *Z* = 4 within the PDF-Global-Fit after each step; the ranking is based on all other structure candidates in *P*2_1_/*c*

PDF-Global-Fit step	Rank	{S}_{12}^{\rm PDF}	{R}_{\rm wp}^{\rm PDF} (%)	*a* (Å)	*b* (Å)	*c* (Å)	α (°)	β (°)	γ (°)
Comparison (step 2)	146	0.98616	–	7.5630	4.9599	13.9036	90	111.1310	90
SA fit (step 3)	3	0.99205	31.857	7.5264	5.0621	13.5931	90	105.581	90
Automated refinement (step 4)	5	–	42.35†	4.987 (5)	7.622 (6)	14.46 (11)	90	107.59 (7)	90
User-controlled refinement (step 5)	1	–	19.57	4.8439 (16)	8.929 (3)	12.423 (3)	90	107.634 (19)	90

† The increase in the {R}_{\rm wp}^{\rm PDF} value from the SA fit to the automated refinement is due to the extended PDF comparison range from 1–20 Å to 1–30 Å.
