# Supplementary material for: Structure determination of organic compounds by a fit to the pair distribution function from scratch without prior indexing
Source: J Appl Crystallogr. 2021 May 9;54(Pt 3):776–86. doi: 10.1107/S1600576721002569 (PMC8202035; doi:10.1107/S1600576721002569)
Supplement: Supplementary file 1 [file j-54-00776-sup1.pdf]

JOURNAL OF APPLIED CRYSTALLOGRAPHY

Volume 54 (2021)

Supporting information for article:

Structure determination of organic compounds by a fit to the pair distribution function from scratch without prior indexing

Carina Schlesinger, Stefan Habermehl and Dragica Prill

Journal of Applied Crystallography

ISSN 1600-5767

Structure determination of organic compounds by a fit to the pair distribution function from scratch without prior indexing

Carina Schlesinger, Stefan Habermehl and Dragica Prill*

Institute of Inorganic and Analytical Chemistry, Goethe University, Max-von-Laue-Str. 7, 60437 Frankfurt am Main, Germany

See: doi.org/10.1107/S1600576721002569

Table S1 The ten best structural models for each space group $P\ 2_1/c$ and $P\ \bar{1}$ according to the similarity measure from the comparison step (Step 2) of the simulated PDF curve to the experimental one. For comparability the unit cells are transformed to a standard setting.

Structure nr.	Space group	S_{12}^{PDF}	V / Å^3	a / Å	b / Å	c / Å	α / °	β / °	γ / °
74505	$P\ 2_1/c$	0.98867	484.72	6.3715	4.7606	16.4054	90	103.07321	90
63658	$P\ 2_1/c$	0.98857	498.91	3.8486	10.4250	12.7314	90	102.38472	90
14990	$P\ 2_1/c$	0.98807	479.13	5.9166	6.0078	13.4878	90	92.05359	90
53484	$P\ 2_1/c$	0.98798	478.48	4.5207	8.2022	12.9041	90	90.15382	90
14456	$P\ 2_1/c$	0.98795	494.18	3.9002	9.3667	13.7576	90	100.49867	90
10572	$P\ 2_1/c$	0.98793	517.45	4.1244	12.1610	10.3244	90	92.21839	90
12906	$P\ 2_1/c$	0.98787	478.33	3.6950	12.1050	11.2863	90	108.63799	90
10581	$P\ 2_1/c$	0.98787	468.85	7.3272	10.1090	6.8278	90	112.01819	90
54325	$P\ 2_1/c$	0.98786	525.56	6.7867	8.9131	8.7621	90	97.43980	90
63390	$P\ 2_1/c$	0.98783	459.30	5.5010	12.8035	7.1401	90	114.03241	90
75386	$P\ \bar{1}$	0.98903	232.40	3.6098	5.8026	11.2284	81.24075	88.84267	89.62096
31070	$P\ \bar{1}$	0.98899	244.61	3.5951	6.0348	11.2966	87.78534	87.72826	88.21101
6626	$P\ \bar{1}$	0.98880	235.91	4.0848	7.6831	8.2623	103.94298	105.71734	98.46187
68397	$P\ \bar{1}$	0.98872	228.58	3.3679	5.7372	11.9513	82.87853	87.40186	86.61885
65993	$P\ \bar{1}$	0.98857	229.24	3.4630	6.4152	10.8234	74.76291	85.91711	81.36423
41141	$P\ \bar{1}$	0.98818	238.88	4.9278	7.1466	7.9689	104.28341	104.65984	109.30319
42426	$P\ \bar{1}$	0.98812	265.31	4.2914	6.6802	9.6299	103.28189	95.30469	95.92610
50984	$P\ \bar{1}$	0.98800	229.53	3.6442	5.8865	11.5313	103.30882	90.58841	106.90421
52508	$P\ \bar{1}$	0.98785	258.03	3.7690	6.2932	11.1647	91.80456	96.17634	101.04892
32793	$P\ \bar{1}$	0.98784	227.46	3.7578	6.4335	9.5797	86.61523	83.90621	81.37273
